# Ameliorative Effects of Thymoquinone against Chemotherapy-Induced Testicular Damage in Experimental Animals: A Systematic Review

**DOI:** 10.21315/mjms2024.31.4.3

**Published:** 2024-08-27

**Authors:** Sukinah A. Tarmookh, Amal Ahmed El-Sheikh, Kamaluddin H. Motawei, Khulood Mohammed Al-Khater, Sujatha Banglore, Rashid A. Aldahhan

**Affiliations:** Department of Anatomy, College of Medicine, Imam Abdulrahman Bin Faisal University, Dammam, Saudi Arabia

**Keywords:** chemotherapy, thymoquinone, testicular damage, animal model

## Abstract

Chemotherapeutic drugs have demonstrated effectiveness in treating various neoplastic conditions; however, they can also have detrimental effects on male gonadal function and fertility. Consequently, interest has grown in identifying novel approaches that can mitigate chemotherapy-induced testicular damage. Thymoquinone (TQ), the chief active component of the volatile oil of *Nigella sativa* (NS), has a wide range of therapeutic properties, including antioxidant, anti-inflammatory and anti-apoptotic effects. The aim of this systematic review was to identify experimental animal studies that have evaluated the protective effects of TQ against testicular complications associated with chemotherapy. In accordance with the preferred reporting items for systematic review and meta-analyses (PRISMA) guidelines, a thorough search was performed across several databases (PubMed, EBSCOhost, Sage and Scopus) to identify experimental studies published from 2010 to May 2022 that focused on rodent models and compared the effects of TQ versus other chemotherapeutic drugs. Eight studies met the inclusion criteria, comparing TQ with methotrexate (MTX), 6-mercaptopurine (6-MP), cyclophosphamide (CPA), bleomycin (BL), doxorubicin (DOX) or busulfan (BUS). The results of these studies consistently demonstrated that TQ significantly improved sperm parameters, the levels of oxidative stress (OS) markers, apoptosis markers, and hormones and testicular histopathology, indicating that TQ has protective effects against chemotherapy-induced damage. TQ mitigated chemotherapy-induced testicular toxicity by decreasing lipid peroxidation and enhancing the activity of antioxidant enzymes within chemotherapy-treated testes. These findings highlight the potential of TQ as a therapeutic agent that can ameliorate testicular complications associated with chemotherapy, thereby providing a basis for further research and potential therapeutic applications.

## Introduction

Chemotherapy drugs are essential components of testicular cancer treatment; however, they are known to cause azoospermia or oligozoospermia (1). This adverse action occurs through the disruption of DNA synthesis and RNA transcription in cancerous cells and affects the rapidly dividing testicular germinal epithelium (1). While chemotherapeutic agents eliminate hyperproliferative malignant cells by targeting genetic material (2), they also attack rapidly dividing cells with a lack of specificity (that is, they target any cell undergoing an active proliferative cycle) (2). Anticancer medications (such as 6-mercaptopurine [6-MP], methotrexate [MTX] and busulfan [BUS]) noxiously affect male reproductivity, causing reduced cellular content and sperm parameters, histopathological changes and increases in apoptotic cell numbers in rat and mouse testes (3–7). Mechanistically, chemotherapy agents initiate this damage by a pro-apoptotic mechanism that produces reactive oxygen species (ROS), lipid peroxidation and DNA fragmentation (3–5). This response triggers oxidative stress (OS), thereby impairing testicular function and human fertilisation potential (8). For this reason, chemotherapy treatments may be reduced or discontinued, which interferes with therapeutic goals. Therefore, an effective parallel preventive plan that minimises testicular toxicity from chemotherapy is essential.

Herbal medicine, as an alternative treatment, plays a significant role in managing multiple diseases (9, 10). One herbal agent widely used in traditional European and Asian medicine is *Nigella sativa* (NS, also known as black seed or black cumin), which contains thymoquinone (TQ, 2-isopropyl-5-methylbenzo-1, 4-quinone) as the chief active component of its volatile oil (11, 12). The therapeutic consequences of TQ administration after exposure to reactive chemotherapeutic compounds have recently become an area of interest in several conventional medical studies (13, 14). TQ protects against doxorubicin-induced cardiotoxicity without affecting the anticancer activity of the drug in mice (15), and it attenuates cisplatin-induced nephrotoxicity and doxorubicin-induced nephrotoxicity in rats (16, 17). TQ acts as a superoxide radical scavenger and eliminates free radicals or ROS by regulating the oxidant/antioxidant balance (18). TQ also modulates the chemotherapy-induced deterioration of various cell types and restores tissue integrity in rats (13). These experimental findings have prompted the examination of the healing properties of TQ in several different animal models.

Although several researchers have reported the efficacy of TQ in treating chemotherapeutic-induced toxicity in various organs, few studies have examined the potential protective effects of TQ against chemotherapy-induced testicular disorders. The aim of this systematic review was to evaluate published experimental investigations that have explored the ameliorative effects of TQ against chemotherapy-induced testicular damage in rodent models. The analysis includes an evaluation of the therapeutic potential of TQ after testicular disruption by commonly used anti-neoplastic agents, namely MTX, 6-MP, cyclophosphamide (CPA), bleomycin (BL), doxorubicin (DOX) and BUS.

## Methods

### Search Strategy

This systematic review was planned according to the preferred reporting items for systematic review and meta-analyses (PRISMA) guidelines (19). The PubMed, EBSCOhost, Sage and Scopus databases were searched to generate the references for this systematic review. All databases were searched using keywords combined with synonyms, truncations and Boolean operators. The literature search was performed using the following search terms in the titles and abstracts: [thymoquinone OR *Nigella sativa* OR black seed OR black cumin] AND [testis OR testicular damage OR testicular OR male reproductive OR male germ cell OR male gonad OR germ cell toxicity OR spermotoxicity OR male germ cell abnormalities OR male gonad alterations] AND [chemotherapy OR antineoplastic agents OR cancer treatment] AND [rats OR mice OR animal model]. The reference lists of the incorporated studies were also searched for additional potentially eligible articles.

### Eligibility Criteria

A precise framework to guide the inclusion and exclusion criteria was defined for this systematic review. The literature included in this systematic review met the following criteria: full-text original articles published between 2010 and May 2022; written in the English language; investigating a research question following the population, intervention, comparison, and outcome (PICO) format; and conducted on animals with comparison to a control group. Review articles, human studies, case reports, studies with no comparison data, studies with inappropriate outcomes and uncontrolled studies were excluded.

### Study Selection

Three reviewers (SAT, AAE and RAA) assessed the eligibility of each article to minimise the risk of bias by screening the title and abstract, and studies that failed to satisfy the inclusion criteria at this phase were excluded. Subsequently, the full text of each carefully chosen paper was evaluated and the data were extracted. The fourth reviewer (KHM) was consulted in case of any conflicts.

**Figure 1:**
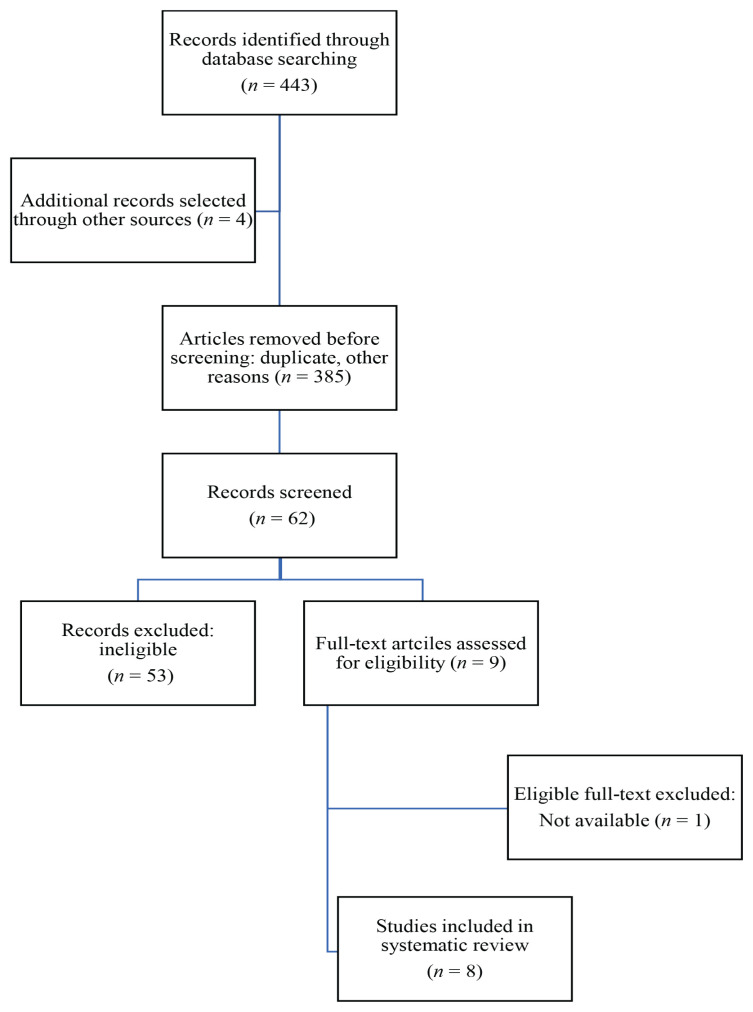
PRISMA flow diagram demonstrating the article selection process

Studies evaluating the impact of NS oil as a protective agent were excluded because this systematic review focused on thymoquinone, the main active biochemical compound of NS oil and not the plant itself or its oil. Many studies appeared more than once in multiple databases; thus, removing duplicate studies was crucial to prevent repetition. Overall, the final number of accepted articles for this systematic review was eight (Figure 1).

### Data Collection

The data extracted from the articles included the following sections: descriptive information about the article, study design features, animal species and sex, drug doses, drug administration route, duration of drug treatment and outcome results (such as histological, statistical analysis and genetic examinations). The reviewers then compared these sections to identify the similarities and differences among the accepted articles. Detailed information on the data extracted for all categories is presented in Table 1.

### Risk of Bias

The Systematic Review Centre for Laboratory Animal Experimentation (SYRCLE), a risk of bias tool for animal study, was used to evaluate potential bias in the included articles (20). This tool is based on the Cochrane risk of bias tool (21) and has been adjusted for bias features in animal interference studies. The tool utilises five main categories of bias: reporting bias (selective outcome reporting), selection bias (allocation concealment, sequence generation and baseline characteristics), attrition bias (incomplete outcome data), detection bias (random outcome assessment and blinding) and performance bias (random housing and blinding).

## Results

The PICO framework guided the inclusion criteria for the accepted studies. The population of interest in the selected studies was male animals that had suffered from testicular injury associated with chemotherapeutic medication. The studies examined the impact of TQ as a medical intervention that could protect against chemotherapeutic drug-induced toxicities. The protocols of the experiments utilised various designs and statistical approaches to reach the study outcomes.

### Search Results

The PRISMA flow diagram of the article search is presented in Figure 1. The initial search identified 443 titles and abstracts, with four additional studies identified from the reference lists of other studies. Of these, 385 were excluded as duplicates and for other reasons (such as language different from English, non-original studies). We then screened the remaining 62 titles and abstracts for eligibility. Of these, we excluded a further 53, leaving nine potentially eligible studies. These were read in full, and one article was excluded because of restricted access. In total, eight original published articles were included in this review (3–7, 22–24). Four of these studies were performed in Iran, two in Turkey, one in Egypt and one in Malaysia.

### Study Characteristics

The characteristics of the involved articles are presented in Table 1. The incorporated studies represent experimental studies with lucid aims, methods, outcomes and discussion. The type of chemotherapeutic drugs used and their administration methods varied among the studies, with seven studies employing intraperitoneal injections and one study administering the drugs via intragastric gavage. The administered doses of TQ ranged from 5 mg/kg to 20 mg/kg for periods ranging from 14 days to 53 days. Two experimental animal species were studied: mice and rats. Five studies used different mouse strains (C57Bl/6 [*n* = 1] and BALB/c [*n* = 4]), and three Wistar albino rats.

### Sperm Parameters

Chemotherapy administration noticeably lowered sperm motility, count and viability while also increasing abnormal morphology. All these parameters showed significant improvement with the co-administration of TQ (3, 5–7, 22, 24) (Table 2).

### Oxidative Stress and Inflammatory Biomarkers

Experimental studies have reported that TQ has protective effects on the testicular structure against the OS that occurs due to the deleterious effects of chemotherapy (Table 2). The level of malondialdehyde (MDA), an indicator of OS, was increased, whereas the levels of antioxidant markers, such as total antioxidative capacity (TAC), glutathione (GSH), total oxidative stress (TOS), catalase, heat shock proteins (HSP) and myeloperoxidase (MPO), were decreased in chemotherapy-treated rats (3, 5, 23) and mice (4, 24). Testicular TNF-α was significantly higher in the chemotherapy-treated group of rats than in the control group. However, the group administered the combined chemotherapy and TQ treatment showed a marked decline in TNF-α (3).

### Apoptosis Markers

The mRNA expression of pro-apoptotic marker genes caspases 3, 8 and 9; p53 and Bax was significantly upregulated compared, while Bcl-2 and Pi3k mRNA expression was substantially downregulated in chemotherapy-treated mice. The administration of TQ to chemotherapy-treated mice restored these markers close to the control level (3, 6, 24), with a concomitant minimisation of germ cell apoptosis (Table 2).

### Hormones

The included studies reported that the administration of antineoplastic agents decreased serum testosterone levels (3, 5, 23, 24) and elevated serum follicle-stimulating hormone (FSH) and luteinising hormone (LH) levels (24). The TQ co-therapy maintained serum testosterone, FSH and LH levels at the control levels. The expression of *Ar* mRNA was significantly lower in 6-MP-treated rats than in rats co-administered with TQ and 6-MP (3) (Table 2).

### Histopathological Results

Antineoplastic treatment causes severe disruption of the germinal epithelium and interstitium (Table 3). Specifically, chemotherapy administration induced a significant decrease in the testicular weight of rats (3, 23) and mice (24), but this decrease was considerably minimised in TQ-treated groups. Two studies reported a significant decrease in seminiferous tubule (ST) diameter after DOX administration to rats (23) and BL to mice (24), while one study demonstrated an increase in diameter after CPA administration to mice (22). The ST diameter was maintained by the co-administration of TQ.

Most of the studies reported various degrees of degenerative testicular changes after chemotherapy treatment, including desquamation of germ cells from the basal lamina with severe disruption of the germinal epithelium, thickened tunica albuginea, interstitial space oedema, Leydig cell atrophy, decreased numbers and sizes of almost all spermatogenetic cells and the presence of cytoplasmic vacuolisation. TQ exerts a protective effect by minimising the abnormalities caused by chemotherapy treatment (3–5, 22–24). Chemotherapy treatment increased the immunostaining of caspase 3 and Hsp90 immunostaining in germinal cells, but the increase in immunoreactivity was suppressed by co-therapy with TQ (23). Terminal deoxynucleotidyl transferase dUTP nick end labelling (TUNEL) staining of testicular tissue also showed a much greater increase in the apoptotic cell number in the chemotherapy groups than in the groups treated with the combined chemotherapy and TQ regimen (6, 23) (Table 3).

### Risk of Bias in the Included Studies

**Figure 2:**
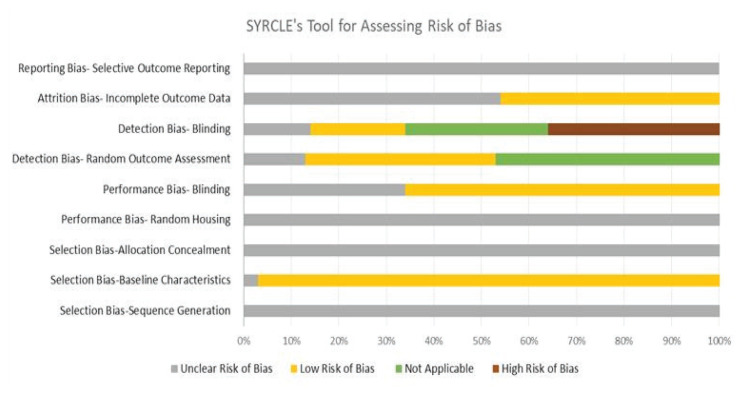
SYRCLE risk of bias tool used to detect the bias score of accepted articles in this systematic review. Low risk indicates an accurate bias alleviation. High risk of bias indicates a strong sign of possible bias. Not applicable bias means that the cause of bias was not significant. Unclear risk indicates that the studies did not mention any alleviation of bias. The score (%) represents the risk of bias for each section of the tool

The SYRCLE risk of bias tool measures the bias score of each paper to provide validity (Figure 2). The primary finding from the risk of bias and methodological quality assessments is the prevalence of ‘unclear’ scores, indicating inadequate reporting in many aspects (for example, none of the studies described details of random housing, allocation concealment, and sequence generation). While several studies indicated that animals were randomly assigned to the house, the specific randomization method employed needed to be clarified. Most of the studies mentioned detailed baseline characteristics of the animals (such as sex, age and weight), showing a low risk of bias. Regarding detection bias, some studies were scored with a low risk of bias on these items and described as random outcome assessments. In addition, a high risk of bias was identified in some studies that did not report sufficient blinding of detection bias. With respect to attrition bias, some studies effectively addressed the issue of incomplete outcome data (low risk of bias). In contrast, others received an unclear risk of bias score in this regard. Concerning reporting bias, none of the studies included in the analysis registered their protocols on a publicly accessible registry platform, making it unclear whether selective reporting bias was present.

## Discussion

This systematic review evaluated published studies that examined the impairments induced by chemotherapeutic agents (MTX, CPA, 6-MP, DOX and BUS) and the ameliorative effects of co-administration of TQ on the testes of rats and mice. Although chemotherapeutic drugs are highly effective in managing many types of malignancies, their use is restricted because of their serious dysfunctional side effects on numerous organs, particularly vital reproductive organs (25).

The use of protective agents during chemotherapy has been suggested as a feasible way to decrease chemotherapeutic side effects (11). Protective agents like TQ have been used as antioxidant, antiapoptotic, antitumour and anti-inflammatory agents (18). The present review indicates that a combination of TQ and chemotherapeutic drugs could have beneficial therapeutic consequences in diminishing drug toxicity to the testes. The following are potential mechanisms that might be involved.

Semen analysis is a routine part of evaluating male fertility in clinical practice (26). The effect of TQ on sperm motility depends on the dosage, with high doses exhibiting a spermostatic effect (27). In the papers reviewed here, TQ prevented chemotherapy-induced reductions in sperm count, motility, viability and morphology. These findings align somewhat with previous studies on TQ in different infertile experimental mouse models, specifically in morphine-treated and ageing model mice (28, 29) and in rats exposed to an organophosphate pesticide (diazinon) or induced to become diabetic (30, 31). The findings are also consistent with a double-blind randomised study showing that administration of NS oil for 2 months had a normalising effect on various parameters, such as sperm structure, locomotion, semen acidity and semen volume, in infertile men (32).

The findings of this review demonstrated a decline in testosterone levels and an increase in FSH and LH levels following chemotherapy. However, these hormones were maintained at their control levels by the co-administration of TQ. These data are in agreement with the findings of Alyoussef and Al-Gayyar (33), who reported that the co-application of TQ to rats maintains the balance of sex hormones and FSH and LH levels while preventing the decrease in testosterone induced by sodium nitrite treatment. Similarly, losses of testosterone in nicotine-treated rats were significantly mitigated by TQ treatment (34). These results support the marked effect of TQ in preventing the reduction in plasma testosterone levels observed in rats following lead administration (35). The decline in testosterone levels and in *Ar* mRNA expression is ascribed to the chemotherapy-induced atrophy of Leydig cells, which is suppressed by the co-administration of TQ, as evidenced by the maintenance of normal levels of testosterone and *Ar* mRNA gene expression (3).

Chemotherapy administration in the included studies of this systematic review clearly distorted testes histology, as indicated by the detachment of germ cells from the basal lamina, necrosis in almost all STs, degenerated Leydig cells and reduced spermatocyte density (Table 3). Desquamation of germ cells from the basal lamina results in the separation of Sertoli cells from spermatogonia and prevents their interaction, with a consequent decline in the proliferation of spermatogonia and a reduction in sperm count (36). Öztürk and colleagues (23) attributed the histopathological changes observed in the testes to germ cell apoptosis, as determined using the TUNEL technique. Apoptosis of spermatogenic cells after chemotherapy exposure is induced by OS and leads to defective sperm chromatin integrity and DNA fragmentation (22). The histopathological changes were mitigated in the testes of TQ-treated rats and mice (3–5, 22), as well as in TQ-treated animals subjected to other damaging agents, such as lead (35), cadmium (37) and torsion-induced oxidative injury (38). Significant suppression of structural damage to the testes can be achieved through the ability of TQ to decrease the activity of Bax and caspase-3 apoptotic pathways (39) and prevent ROS generation (40).

The reviewed studies here showed a significant increase in OS, resulting in excessive release of ROS and MDA, along with decreased activities of the antioxidant enzymes GSH, TAC, TOS, HSP, MPO and catalase. Spermatogenic cells are highly susceptible to lipid peroxidation and OS as a result of their increased concentration of polyunsaturated fatty acids (41). Chemotherapeutic drugs increase OS in the environment surrounding germinal cells and induce apoptosis of spermatogenic cells (3–5, 24). The anti-apoptotic effect of TQ could be mediated by an increase in anti-apoptotic Bcl2 gene expression and a reduction in the expression of pro-apoptotic Bax, p53 and caspase 3 (6, 24). The extrinsic and intrinsic apoptosis pathways activate their initiator caspases 8, 9 and 10 to eventually activate the executioner caspase 3 (42). The level of caspase 3 in chemotherapy-treated animals was increased and caused cytoskeletal rearrangement and breakdown of the cell into apoptotic bodies (3, 23, 43). Some studies also support an anti-apoptotic mechanism for TQ effects in rat testicular ischemia-reperfusion injury (44), a myocardial infarction rat model (45) and sepsis-induced cardiac damage in mice (46).

One of the studies included in this review revealed that 6-MP increased the levels of inflammatory cytokines and tumour necrosis factor (TNF-α), indicating chemotherapy-related inflammation in testicular tissues. However, co-administration of TQ with 6-MP significantly suppressed the increase in TNF-α level, suggesting that TQ has anti-inflammatory effects (3). This finding is in agreement with the observed reductions in levels of various pro-inflammatory regulators, including interleukin 1-beta (IL-1β), interleukin 6 (IL-6) and TNFα, in TQ-treated rats (47). Nevertheless, the timing of these changes in pro-inflammatory markers was not determined, which raises a question regarding the resultant inflammation type (acute or chronic). TQ also exerts its anti-inflammatory and antioxidant effects through multiple molecular pathways, including the release of cytokines and activation of signalling pathways involving cyclooxygenase-2 (COX-2), nuclear factor erythroid 2-related factor 2 (Nrf2), phosphatidylinositol 3-kinase/protein kinase B (PI3K/AKT) and nuclear factor kappa-light-chain-enhancer of activated B (NF-κβ) (48). Additional studies have provided further evidence of the anti-inflammatory effects of TQ in different rodent models, such as cardioprotective activity in myocardial ischaemic injury, an anti-arthritic effect on arthritis, an inhibitory effect on neuroinflammation in microglia in rats (49–51) and a palliative effect on allergic lung reactions in mice (52).

### Limitations

This systematic review has some limitations that need to be recognised. One is the small number of included articles, as well as the exclusion of some eligible studies due to insufficient recording of the outcome data or restricted access. In addition, the studies in this review were heterogeneous in many aspects, including variances in the sample population in terms of the races and ages of the animals and differences related to the administration of drugs, dosages and durations of treatment; all of these differences could lead to incorrect and unreliable outcomes. The evaluated reports also lack any specific examination—either quantitative or qualitative—of the supporting somatic, Sertoli or Leydig cells, even though these cells play known and crucial roles in testes function (53). The hypothalamic-pituitary-testicular (HPT) axis was also not considered in these studies. Although the serum levels of gonadotropins were reported, a thorough discussion of the components of the HPT axis is essential. The reviewed studies failed to report the effect of anti-cancer therapies and TQ on inhibin, a spermatogenesis marker and regulatory factor of FSH secretion (54). The mechanism by which TQ minimises the testicular toxicity of chemotherapy remains ill defined, since most studies observed a protective effect without characterising the actual molecular processes that were impacted.

### Future Research Needs

The findings evaluated in this review highlight the need for further experimental research that embraces multiple approaches when evaluating chemoprotective drugs. Despite the promising results outlined by the co-administration of chemotherapy and TQ drugs, the evidence provided by animal studies needs to be revisited for transfer to clinical trials and needs more research. Further mechanistic research is also necessary to understand how chemotherapy drugs target the testes at different developmental stages and to determine dosages and administration schedules for chemoprotective drugs that could potentially prevent this damage. An examination of many different chemotherapeutic drugs and other biochemical parameters is also necessary to obtain better information on the protective effects of TQ.

## Conclusion

This systematic review delivers a summary of the therapeutic effects of TQ in preventing chemotherapy-induced testicular destruction in animal models. As an anti-inflammatory, anti-oxidant and anti-apoptotic component of a known therapeutic herb, TQ may improve chemotherapy-induced fertility impacts by maintaining sperm health and minimising apoptosis, OS and testicular degeneration.

## Figures and Tables

**Table 1:** Summary of the characteristics of the eligible studies investigating thymoquinone’s role in alleviating testicular damage induced by chemotherapeutics

References	Country	Rodent species	Age/weight of rodents	Number of rodents/groups	Follow-up (day)	Control group treatments	Chemotherapy	Route of administration	Funding
Gökce et al. (4)	Turkey	Male C57BL/6 mice	6 weeks**/**20 + 2 gm	24/4	42	Saline	Methotrexate	Intraperitoneal	No specific grant from any funding agency
Kamarzaman et al. (22)	Malaysia	Male BALB/c mice	6–8 weeks**/**20–30 gm	60/4	53	Vehicle	Cyclophosphamide	Intraperitoneal	Grant from the International Islamic University Malaysia
Sheikhbahaei et al. (6)	Iran	Male BALB/c mice	10 weeks/30 ± 2 gm	30/5	16	Saline	Methotrexate	Intraperitoneal	Grant from Kermanshah University of Medical Sciences
Shikhbahaei et al. (7)	Iran	Male BALB/c mice	Not available/25–30 gm	30/5	16	Dimethyl sulfoxide in saline	Methotrexate	Intraperitoneal	Grant from the Kermanshah University of Medical Science Research
Abdelbaky et al. (3)	Egypt	Male albino rats	Not available/180–200 gm	40/4	30	Saline	6-mercaptopurine	Intragastric gavage	Grant from the Scientific Research Developing Center in Benisuef University
Öztürk et al. (23)	Turkey	Male Wistar albino rats	9 weeks/200–250 gm	40/5	14	Olive oil	Doxorubicin	Intraperitoneal	Grant from the Research and Technology Department of Erciyes University
Jalili et al. (5)	Iran	Male Wistar rats	8 weeks/220–250 gm	64/8	35	Saline	Busulfan	Intraperitoneal	Not mentioned
Yaghutian et al. (24)	Iran	Male BALB/c mice	8 weeks/30 ± 2 gm	48/6	35	Saline	Bleomycin	Intraperitoneal	Grant from the Shiraz University of Medical Sciences

**Table 2:** Comprehensive analysis of blood serum markers, testicular tissue markers, and sperm parameters in assessing the ameliorating effects of thymoquinone on chemotherapeutics-induced testicular damage

Author	Chemotherapy dosage	Outcome of testicular impairment by chemotherapy	Thymoquinone dosage	Thymoquinone administration outcome
Gökce et al. (4)	MTX 20 mg/kg	↑ TAC, ↑ MPO ↑ TOS MDA did not differ among the groups	10 mg/kg	↓ TAC, ↓MPO ↓ TOS
Kamarzaman et al. (22)	CPA 200 mg/kg	↑ DNA fragmentation in spermatozoa, ↓number of elongating spermatids and pachytene spermatocytes	10 mg/kg	↓ DNA fragmentation in spermatozoa, no significant recovery of the spermatids and spermatocytes number
Sheikhbahaei et al. (6)	MTX 20 mg/kg	↓ Bcl2, ↑ p53, ↑ caspase 3, ↑ caspase 8, ↑ caspase 9, ↑ Bax, and Bax/Bcl2	2, 10, 20 mg/kg	↓ Bcl2, ↓ p53, ↓ caspase 3, ↓ caspase 8, ↓ caspase 9, ↓ Bax and Bax/Bcl2
Shikhbahaei et al. (7)	MTX 20 mg/kg	↓ Sperm viability, motility and count	2, 10, 20 mg/kg	↑ Sperm viability, motility and count
Abdelbaky et al. (3)	6-MP 5 mg/kg	↓ Sperm count, motility and viability ↑ Sperm abnormality rate ↓ Testosterone ↑ MDA, ↓ GSH and catalase ↑ caspase-3 and P53 mRNA gene. ↓ PI3K ↑ TNF-α ↓ AR mRNA	5 mg/kg	↑ Sperm count, motility and viability ↓ Sperm abnormality rate ↑ Testosterone ↓ MDA, ↑ GSH and catalase ↓ caspase-3 and P53 mRNA gene ↑ PI3K ↓ TNF-α ↑ AR mRNA
Öztürk et al. (23)	DOX 15 mg/kg	↓ Testosterone ↓ TAS, ↑ TOS ↑ apoptotic cell	10 mg/kg	↑ Testosterone ↑ TAS, ↓ TOS ↓ apoptotic cell
Jalili et al. (5)	BUS 10 mg/kg	↓ Sperm viability, motility and count ↓ Testosterone ↑ MDA ↓ TAS	4.5, 9 and 18 mg/kg	↑ Sperm viability, motility and count ↑ Testosterone ↓ MDA ↑ TAS
Yaghutian et al. (24)	BL 10 mg/kg	↑ LH and FSH ↓ Testosterone ↑ MDA ↓ Sperm viability, motility and count ↓ Bcl2l1 ↓ Bcl2l1/Bax	7.5 and 15 mg/kg	↓ LH and FSH ↑ Testosterone ↓ MDA ↑ Sperm viability, motility and count ↑ Bcl2l1 in BL + TQ7.5 group ↑ Bcl2l1/Bax

Notes: ↑ = increased; ↓ = decreased; MPO = myeloperoxidase; MDA = malondialdehyde; DNA = deoxyribonucleic acid; TOS = total oxidative stress; TAC = total antioxidant capacity; PI3K = phosphoinositide 3-kinase; Bcl2 = B-cell lymphoma 2; Bax = Bcl-2-associated X protein; p53 = tumour protein; TNF-α = tumour necrosis factor-alpha; GSH = glutathione; FSH = follicle-stimulating hormone; LH = luteinising hormone; caspases = cysteine-aspartic proteases; AR = androgen receptor; Bcl2l1 = BCL2-like 1

**Table 3:** Summary of testicular histopathological findings demonstrating thymoquinone’s protective role against chemotherapeutics-induced damage

Author	Chemotherapy administration outcome	Thymoquinone administration outcome
Gökce et al. (4)	Severe disruption of the seminiferous epithelium, interstitial space widening and oedema, ↓ cell size and swelling cytoplasm, moderate thickening of tubular basement membrane	Improved seminiferous tubules morphology
Kamarzaman et al. (22)	↑ diameter of seminiferous tubules, ↑ tubular lumen, degeneration of spermatogenic layers, vacuolisation of spermatogonia, degenerated interstitial tissues, marked decrease of spermatozoa	↓ diameter of seminiferous tubules, ↓ tubular lumen, Most of the seminiferous tubules restored to their normal structure
Sheikhbahaei et al. (6)	↑ TUNEL-positive apoptotic cells	↓ TUNEL-positive apoptotic cells
Shikhbahaei et al. (7)	Not reported	Not reported
Abdelbaky et al. (3)	↓ Testicular weight Desquamation of germ cells from the basal lamina, narrowing of seminiferous tubules, widening in interstitial spaces, vacuolated cytoplasm, Leydig cell atrophy, and degeneration of germ cells and ↓ spermatozoa	↑ Testicular weight Normal histological appearance of testis
Öztürk et al. (23)	↓ Body weight, ↓ testicular weight, ↓ ST diameter, ↓ germ cells count ↓ Seminiferous tubule diameter. Disorganised seminiferous tubule basement membrane, vacuolisation and immature germinal epithelial cells ↑ Caspase 3 and HSP90 immunoreactivities ↑ TUNEL-positive apoptotic cells	↑ Body weight, ↑ testicular weight, ↑ ST diameter, ↑ germ cell count Improvement in testicular histological view ↓ Caspase 3 and HSP90 immunoreactivities ↓ TUNEL-positive apoptotic cells
Jalili et al. (5)	↓ Height of the germinal layer Vacuolisation and reduce sperm cells density	↑ Height of the germinal layer Improved testicular structure
Yaghutian et al. (24)	↓ Testis weight/body weight ratio ↓ Diameters of seminiferous tubules ↑ Thickness of tunica albuginea ↓ Number of spermatogonia, spermatids and Sertoli cells Necrosis in almost all seminiferous tubules	↑ testis weight/body weight ratio ↑ diameters of seminiferous tubules ↓ thickness of tunica albuginea ↑ number of spermatogonia, spermatids and Sertoli cells A lower dose of TQ improved testicular structure, while a high dose of TQ had less effect on restoring injured testicular tissue

Notes: ↑ = increased; ↓ = decreased; TUNEL = terminal deoxynucleotidyl transferase dUTP nick end labeling; ST = seminiferous tubule; HSP90 = heat shock protein 90
